# Prophage Gene *Rv2650c* Enhances Intracellular Survival of *Mycobacterium smegmatis*

**DOI:** 10.3389/fmicb.2021.819837

**Published:** 2022-01-17

**Authors:** Xiangyu Fan, Zichen Liu, Zhibin Wan, Hanlu Zou, Mengzhi Ji, Kaili Sun, Rongfeng Gao, Zhongfang Li, Wu Li

**Affiliations:** ^1^Country College of Biological Science and Technology, University of Jinan, Jinan, China; ^2^School of Life Sciences, Neijiang Normal University, Neijiang, China; ^3^College of Food and Bioengineering, Hezhou University, Hezhou, China; ^4^Guangxi Key Laboratory of Health Care Food Science and Technology, Hezhou University, Hezhou, China

**Keywords:** *Mycobacterium*, pathogenic mechanism, prophage, Rv2650c, intracellular survival

## Abstract

**Background:**

Induced by the pathogen *Mycobacterium tuberculosis*, tuberculosis remains one of the most dangerous infectious diseases in the world. As a special virus, prophage is domesticated by its host and are major contributors to virulence factors for bacterial pathogenicity. The function of prophages and their genes in *M. tuberculosis* is still unknown.

**Methods:**

*Rv2650c* is a prophage gene in *M. tuberculosis genome*. We constructed recombinant *Mycobacterium smegmatis* (*M. smegmatis*) to observe bacteria morphology and analyze the resistance to various adverse environments. Recombinant and control strains were used to infect macrophages, respectively. Furthermore, we performed ELISA experiments of infected macrophages.

**Results:**

Rv2650c affected the spread of colonies of *M. smegmatis* and enhanced the resistance of *M. smegmatis* to macrophages and various stress agents such as acid, oxidative stress, and surfactant. ELISA experiments revealed that the Rv2650c can inhibit the expression of inflammatory factors TNF-α, IL-10, IL-1β, and IL-6.

**Conclusion:**

This study demonstrates that the prophage gene *Rv2650c* can inhibit the spread of colonies and the expression of inflammatory factors and promote intracellular survival of *M. smegmatis*. These results build the foundation for the discovery of virulence factors of *M. tuberculosis*, and provide novel insights into the function of the prophage in *Mycobacterium*.

## Introduction

According to the 2020 global tuberculosis (TB) report released by WHO (World Health Organization [WHO], 2020), about 2 billion people were latently infected by *Mycobacterium tuberculosis* and 1.41 million people had fallen ill with TB in 2019. There were about 1.21 million tuberculosis deaths among HIV-negative people and an additional 208 thousand deaths among HIV-positive people. Tuberculosis is still one of the most prevalent and deadly infectious diseases in the world. Therefore, the pathogenesis and virulence factors of *M. tuberculosis* are still need to be studied. Currently known virulence factors of *M. tuberculosis* can interfere with phagosome maturation (Jayachandran et al., 2007; Queval et al., 2017); control cell death (Awuh and Flo, 2017); disrupt the production of cytokines (Etna et al., 2014); and can help *M. tuberculosis* resist various harsh environments (Ehrt et al., 2018) to maintain the survival in macrophages. However, most genes encoding virulence factors of *M. tuberculosis* are present in the genome of *Mycobacterium smegmatis* (*M. smegmatis*), whereby such genes from *M. smegmatis* can replace their homologous genes in *M. tuberculosis* and cause disease (Cambier et al., 2014). *M. smegmatis* is a kind of non-pathogenic environmental saprophytic mycobacteria. Hence, this raises the question of whether there are any unknown specific virulence factors that play a key and irreplaceable role in the pathogenesis and intracellular survival of *M. tuberculosis*.

As domesticated phage elements that have been integrated into and replicated with the host genome, prophages are involved in the evolution of some bacteria. Prophages can regulate host behavior to improve the host’s fitness to harsh environments. For pathogens, prophages can confer or enhance bacterial virulence and increase the intracellular survival rate and pathogenicity of bacteria (Feiner et al., 2015; Salmond and Fineran, 2015; Argov et al., 2017; Keen and Dantas, 2018). There are two prophages, phiRv1 and phiRv2, in the *M. tuberculosis* H37Rv genome. They do not exist in the *M. smegmatis* genome and their functions in the *M. tuberculosis* genome are still unknown. Our previous study found that some genes of prophage phiRv1 and phiRv2 can be encoded from proteomic data (Deng et al., 2013; Schubert et al., 2013). In this study, we focus on the prophage phiRv2 gene *Rv2650c* which was predicted to encode the capsid protein (Kapopoulou et al., 2011). Transcriptomic data indicated that *Rv2650c* is upregulated in the *M. tuberculosis*-infected macrophage model and persister model (Fan et al., 2016). Those data indicates that Rv2650c is a functional protein and may improve the fitness of *Mycobacterium* to environmental stresses. Moreover, bacteriophage P4 capsid protein, Psu, was considered to be related to the pathogenicity of various pathogens (Ghosh et al., 2018). In summary, capsid protein, Rv2650c, may play an important role in the response of *M. tuberculosis* to stress and pathogenicity.

In this study, we construct recombinant *M. smegmatis* overexpressed *Rv2650c* and carry out a series of experiments to investigate the role of *Rv2650c* in the pathogenicity and intracellular survival of *Mycobacterium*.

## Materials and Methods

### Bacterial Strains, Plasmid, and Growth Conditions

*Escherichia coli* (*E. coli*) DH5α, *E. coli* BL21, *M. smegmatis* mc^2^155, and pNIT-Myc plasmid were preserved by our laboratory. *E. coli* was grown in Luria-Bertani (LB) medium and was used for plasmid construction and preservation. *M. smegmatis* mc^2^155 was grown in either Middlebrook (MB) 7H9 broth or on MB 7H9 agar containing 10 g/L glucose, 2% (v/v) glycerol, and 0.05% (v/v) Tween80. When required, kanamycin (25 μg/mL) and cycloheximide (10 μg/mL) were added. Plasmid pNIT-Myc was constructed from shuttle plasmid pNIT-1 (Pandey et al., 2009; Li et al., 2014). In the plasmid, the inducible *nitA* promoter (*PnitA*) controlled the overexpression of expression cassette.

### Construction of Recombinant *Mycobacterium smegmatis*

The *Rv2650c* gene of phage phiRv2 in the *M. tuberculosis* H37Rv genome was synthesized in Tsingke Biological Technology Co., Ltd. and ligated into the plasmid pNIT-Myc vector. The multiple cloning sites on both sides were *Eco*RI and *Apa*I. Recombinant plasmid pNIT-Myc-Rv2650c was constructed (Supplementary Figure 1), transferred into *E. coli* and subjected to extraction. The recombinant plasmid and empty pNIT-Myc vector was then separately transferred into *M. smegmatis* mc^2^155 using the standard electroporation method of mycobacteria (Pandey et al., 2009). The recombinant *M. smegmatis* strains were selected on MB 7H9 agar containing kanamycin. The positive recombinant *M. smegmatis* was selected by colony PCR and sequenced in Tsingke Biological Technology Co., Ltd. The positive clone was named MS_Rv2650c, and the transformed vector strain was named MS_Vec. Western blot was used to detect the positive recombinant strain.

### Cellular Aggregation and Colony Morphology

The recombinant *M. smegmatis* MS_Rv2650c and MS_Vec were inoculated separately to 7H9 medium with 0.05% (v/v) Tween 80 and 28 mM ε-caprolactam and cultured at 37°C until an OD_600_ of 0.6–0.8 was reached. The cultures were kept at room temperature for 30 min and were observed the accumulation and precipitation.

The MS_Rv2650c and MS_Vec strains were inoculated on MB 7H9 plates with or without 0.05% (v/v) Tween 80 and cultured at 37°C for 5–6 days. The growth of culture colonies was observed.

### Scanning Electron Microscope Analysis

The recombinant *M. smegmatis* MS_Rv2650c and MS_Vec were inoculated separately to 7H9 medium with 28 mM ε-caprolactam and cultured at 37°C until the OD_600_ reached 0.8–1.0. Cultures were harvested by centrifugation and the harvested precipitation were rinsed by 0.1 M PBS buffer. Then the precipitation was resuspended in 2.5% glutaraldehyde solution. The processed precipitation was dehydrated in a series of ethanol. After naturally drying, the samples were sprayed with gold (MC1000) and were observed by scanning electron microscopy (SEM) (Hitachi regular-8100).

### Growth Curve and Stress Analysis of the Recombinant *Mycobacterium smegmatis*

The recombinant *M. smegmatis* MS_Rv2650c and MS_Vec were inoculated separately and cultured at 37°C until an OD_600_ of 0.6–0.8 was reached. 28 mM ε-caprolactam was added to induce the expression of protein. The OD_600_ was measured every 4 h for a total of 68 h. The growth curve of recombinant *M. smegmatis* was plotted according to the recorded data.

The growth of recombinant *M. smegmatis* in response to various stressors was measured according to references (Misra et al., 2011). The log phase of recombinant *M. smegmatis* was collected by centrifugation, and the bacteria were washed with MB 7H9 at pH 4.5; MB 7H9 at a final concentration of 3% hydrogen peroxide; and MB 7H9 medium at a final concentration of 2% SDS. The bacteria were resuspended in 5 mL of the corresponding conditions of 7H9 broth, and the final OD_600_ value was controlled at about 0.5. Under the corresponding conditions, the bacteria were cultured and 100 μL of the bacterial liquid was taken at the 0, 3, and 6 h time points to make a ten-fold concentration gradient dilution. The diluted bacteria were inoculated on MB 7H9 plates containing kanamycin and cultured at 37°C for 3–4 days. The number of colonies was counted to determine the average value for drawing. The concentration gradient of each plate was made in three parallels.

### Infection of Macrophages With Recombinant *Mycobacterium smegmatis*

The suspension cultured THP-1 cells were evenly seeded at 1 × 10^6^ cells per well in a 6-well plate, and three replicate wells were taken. The THP-1 cells were differentiated into macrophages in the presence of 100 ng/mL PMA (Sigma, United States) at 37°C under 5% CO_2_. Macrophages were infected with recombinant *M. smegmatis* at MOI = 10:1 (bacteria: macrophages). At 13, 25, 48 h after infection, macrophages were washed twice and then lysed with a final concentration of 0.025% (w/v) SDS solution to release *M. smegmatis*. The cell lysate of each well was diluted by 10 times and then inoculated onto the MB 7H10 plate containing kanamycin. After 3–4 days, the number of colonies on the plate was counted, and the colony forming unit (CFU) was calculated to compare the ability of intracellular survival of recombinant *M. smegmatis* cells.

### Assay for Cytokines

The culture supernatant after infection of THP-1 cells with MS_Rv2650c or MS_Vec was collected at the corresponding time. The concentrations of the tumor necrosis factor-α (TNF-α), 1L-10, IL-1β, and IL-6 in the supernatants were determined using an ELISA kit (eBioscience).

### Statistical Analysis

Data of this study were analyzed with paired *t*-test. Values of p < 0.05 were defined as statistically significant. The error bar in the experiment was expressed as the difference between the three values.

## Results

### Expression of Prophage Gene *Rv2650c* in *Mycobacterium smegmatis*

**FIGURE 1:**
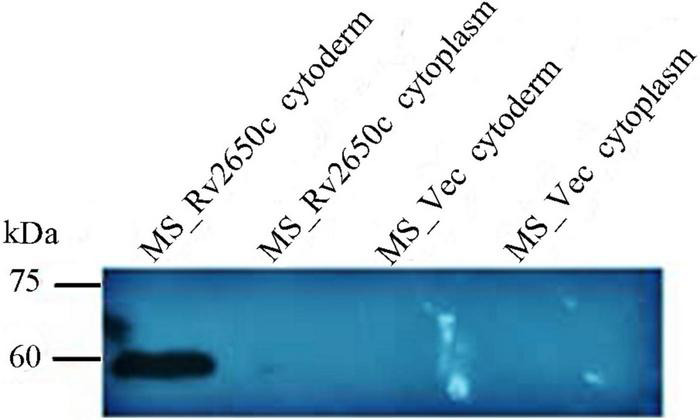
The prophage protein Rv2650c is localized on the cell wall.

The prophage gene *Rv2650c* of *M. tuberculosis* is approximately 1,449 bp in size. We constructed two recombinant *M. smegmatis* to study the function of *Rv2650c*. Among them, the recombinant *M. smegmatis* strain MS_Rv2650c was able to express the *M. tuberculosis* protein Rv2650c carrying the Myc tag. On the contrary, the recombinant *M. smegmatis* strain MS_Vec, which carried the empty plasmid pNIT-Myc, served as the negative control. Both MS_Rv2650c and MS_Vec are capable of expressing the *aph* gene, so both can be grown on kanamycin-containing medium. Western blot analysis confirmed that *Rv2650c* from *M. tuberculosis* was successfully expressed in *M. smegmatis* and localized on the cell wall (Figure 1).

### *Rv2650c* Alters the Diameter Size of *Mycobacterium smegmatis* Colony

**FIGURE 2:**
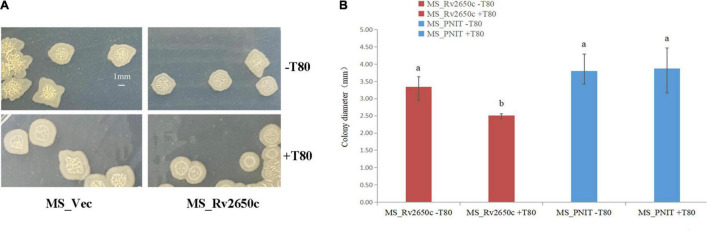
Comparison of the structure of cellular aggregation about the recombinant *M. smegmatis* MS_Rv2650c and MS_Vec. **(A)** MS_Rv2650c and MS_Vec strains were inoculated on MB 7H9 plates with or without 0.05% (v/v) Tween 80 and cultured at 37°C for 5–6 days. **(B)** The diameter of a single colony was observed and counted. Significantly differences (*P* < 0.05) are represented by different letters.

Cell aggregation, single cell morphology, and colony observation experiments were conducted. Comparing the cell aggregation and single cell morphology of MS_Rv2650c and MS_Vec strains, there is no difference between them (Supplementary Figure 2). The result**s** indicated that Rv2650c did not affect the aggregation and morphology of *M. smegmatis*. The results of colony observation experiments showed that the colony diameter of MS_Rv2650c grown on MB 7H9 plates with Tween80 was significantly lower than that of other strains grown on MB 7H9 plates with or without Tween80 (Figures 2A,B). In addition, the edges of colonies of MS_Rv2650c strain grown on the MB 7H9 plates with Tween80 were more rounded while the edges of colonies of other stains were irregular (Figure 2A). The results suggested that the recombinant protein Rv2650c affected the growth and spread of colonies of *M. smegmatis* in the presence of Tween80.

### *Rv2650c* Enhances the Resistance of *Mycobacterium smegmatis* to Various Adverse Environments

**FIGURE 3:**
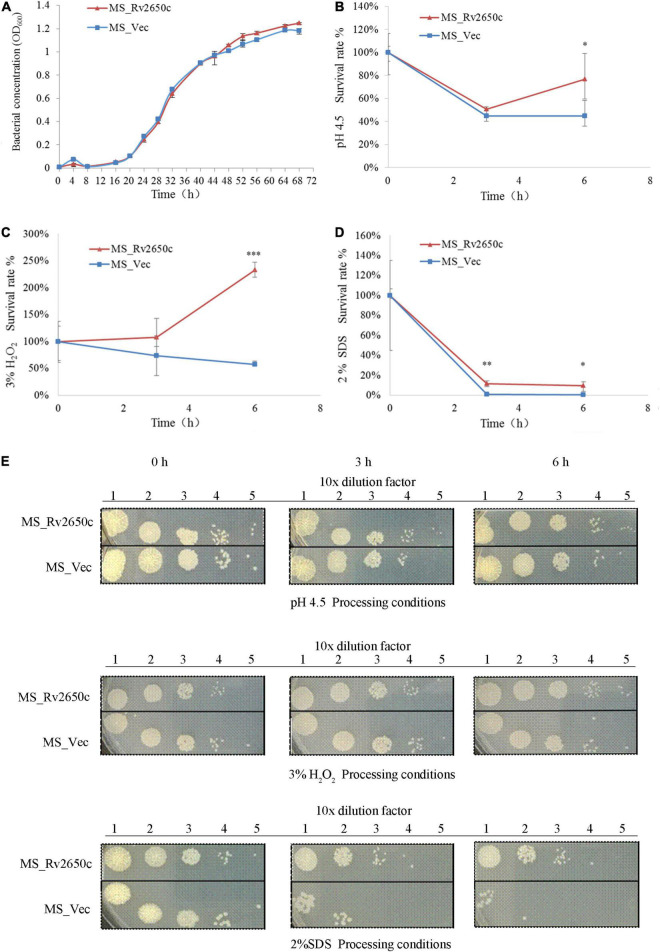
Extracellular growth and stress analysis results of recombinant *M. smegmatis*. **(A)** MS_Rv2650c and MS_Vec were inoculated into MB 7H9 broth and cultured. The OD_600_ value was monitored at 4 h intervals for a total of 68 h. **(B)** Inoculate MS_Rv2650c and MS_Vec into 7H9 broth at pH 4.5 for 6 h at 37°C. The cultured solution of 0, 3, and 6 h was diluted and coated on MB 7H9 plate, and the number of single colonies was calculated after incubation at 37°C for 3–4 days. **(C)** MS_Rv2650c and MS_Vec were treated with 3% hydrogen peroxide at 37°C for 6 h in a cultured solution with an OD_600_ about 0.5, and the cultured solution of 0, 3, and 6 h was diluted and coated on the MB 7H9 plate. The number of single colonies was calculated after incubation for 3–4 days at 37°C. **(D)** MS_Rv2650c and MS_Vec were treated with 2% SDS for 6 h at 37°C in a cultured solution with an OD_600_ about 0.5, and the cultured solution of 0, 3, and 6 h was diluted and coated on the MB 7H9 plate. The number of single colonies was calculated after incubation for 3–4 days at 37°C. **(E)** MS_Rv2650c and MS_Vec were diluted and coated on 7H9 plates under the conditions of pH 4.5, 3% H_2_O_2_, 2% SDS, and cultured at 37°C for 3–4 days. Similar results were obtained in 3 independent experiments. (*p < 0.05; **p < 0.01; ***p < 0.001).

As an intracellular pathogen, *M. tuberculosis* mainly parasitizes in macrophages and faces many complex environments such as acidity, oxidative stress, and poor nutrition. To investigate the role of the prophage protein Rv2650c in the response of *M. tuberculosis* against adverse environmental conditions, we performed growth characteristics analysis of the recombinant *M. smegmatis* MS_Rv2650c and MS_Vec in stress conditions which mimicked the intracellular environment. As shown in Figure 3A, there is no distinct difference in the *in vitro* growth kinetic between two strains, indicating that *Rv2650c* did not change the proliferative activity of *Mycobacterium*. When the recombinant *M. smegmatis* was monitored in MB 7H9 at pH 4.5, the survival rates of MS_Rv2650c and MS_Vec were 76.9% and 44.8% (paired *t*-test, *P* = 0.012), respectively during a 6 h incubation (Figures 3B,E). When cultured in MB 7H9 with hydrogen peroxide (final concentration 3%) for a 6 h incubation period (Figures 3C,E), the survival rates of MS_Rv2650c and MS_Vec were 232.4% and 57.1% (paired *t*-test, *P* = 0.0006). Similarly, MS_Rv2650c had higher rates of survival compared to MS_Vec (11.6% vs. 0.8% after 3 h incubation, paired *t*-test, *P* = 0.008; and 9.3% vs. 0.14% after 6 h incubation, paired *t*-test, *P* = 0.017) when strains were cultured in MB 7H9 with SDS (final concentration 2%) (Figures 3D,E). Taken together, these results showed that the prophage protein Rv2650c can enhance the resistance of recombinant *M. smegmatis* against harsh environments. It also indicates that this ability is not dependent upon improving the replication of MS_Rv2650c strain.

### *Rv2650c* Enhances the Intracellular Survival of *Mycobacterium* in Macrophages

**FIGURE 4:**
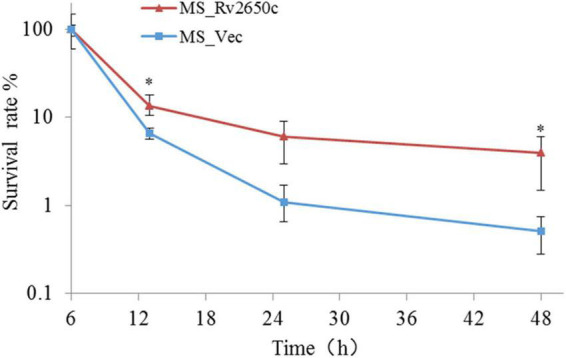
The intracellular survival rate of macrophages infected with recombinant *M. smegmatis*. THP-1 cells were infected with recombinant *M. smegmatis* MS_Rv2650c and MS_Vec, respectively, and the cells were lysed with SDS at the corresponding time. The cell lysate was diluted and coated on MB 7H10 plate. The number of single colonies was calculated after incubation for 3–4 days at 37°C. Similar results were obtained in 3 independent experiments. (*p < 0.05).

Because the *Rv2650c* confers the ability of recombinant bacteria to resist harsh environments, we further studied the intracellular survival of MS_Rv2650c and MS_Vec in macrophages. To achieve this, we infected THP-1-derived macrophages with MS_Rv2650c or MS_Vec as previously described (MOI = 10:1). The experimental results showed that the intracellular survival rate of MS_Rv2650c strain was higher than that of the MS_Vec strain after 13, 25, and 48 h post infection (Figure 4). Rv2650c had significant effect on the intracellular survival of *M. smegmatis* (13.5% of MS_Rv2650c vs. 6.6% of MS_Vec after 13 h post infection, paired *t*-test, *P* = 0.043; 6% of MS_Rv2650c vs. 1.1% of MS_Vec after 25 h post infection, paired *t*-test, *P* = 0.051; 4% of MS_Rv2650c vs. 0.5% of MS_Vec after 48 h post infection, paired *t*-test, *P* = 0.029). This result showed that the expression of *Rv2650c* can enhance the intracellular survival of *M. smegmatis* in macrophages.

### *Rv2650c* Suppresses the Secretion of Pro-inflammatory Cytokines in Macrophages

**FIGURE 5:**
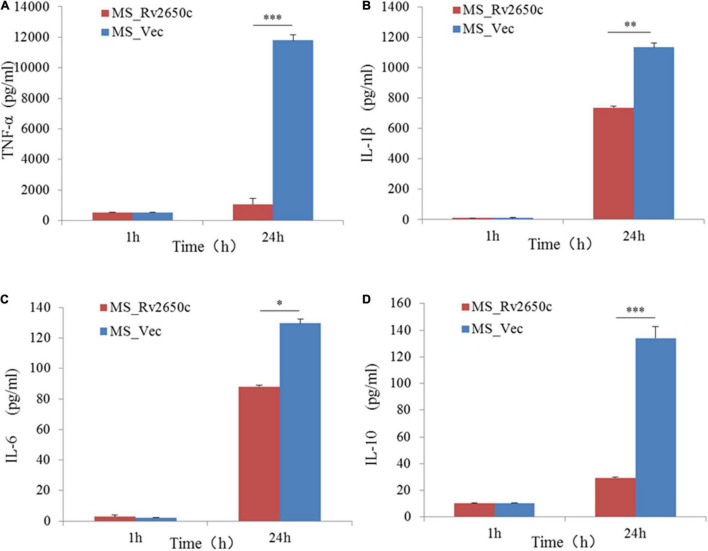
Assay of secretion of cytokines in macrophages. The macrophages were infected with recombinant *M. smegmatis* MS_Rv2650c and MS_Vec at MOI 10:1, respectively, and the secretion of cytokines TNF-α, IL-10, 1L-1β, and 1L-6 in macrophages was measured by ELSA. The supernatant at a certain time point was collected to detect the secretion of TNF-α **(A)**, 1L-1β **(B)**, 1L-6 **(C)**, and IL-10 **(D)**. Similar results were obtained in 3 independent experiments. (*p < 0.05; **p < 0.01; ***p < 0.001).

To detect the effect of the prophage protein Rv2650c on pro-inflammatory cytokines in macrophages, we further tested the expression of some cytokines by ELISA. The results showed that the ability of MS_Rv2650c to stimulate the secretion of cytokines TNF-α, IL-1β, IL-6, and IL-10 was lower than that of MS_Vec after 24 h of infection (paired *t*-test; *P* = 0.000006, *P* = 0.004, *P* = 0.01, *P* = 0.00002, respectively). The results indicated that the expression of Rv2650c significantly inhibited the secretion of pro-inflammatory factors in macrophages (Figure 5).

## Discussion

In this study, we evaluated the function of the prophage gene *Rv2650c* in *M. tuberculosis*. The results of morphological experiments suggested *Rv2650c* affected the spread of colonies of *M. smegmatis* in the presence of Tween80. MS_Rv2650c showed stronger survivability than MS_Vec did, when they infected macrophages and faced adverse environments such as acidity, oxidative stress, and surfactants. Further, ELISA experiments confirmed that *Rv2650c* inhibit the expression of inflammatory factors TNF-α, IL-10, 1L-1β, and IL-6. These results demonstrated that the prophage protein Rv2650c promoted the survival of *Mycobacterium* in macrophages for the first time.

In 1998, it was first revealed that two prophages, phiRv1 and phiRv2, were hidden in the complete genome sequence of *M. tuberculosis* H37Rv (Cole et al., 1998). As black boxes, the significance and role of these two phages remain unclear. Some studies show that prophage is related to the evolution and adaptation of host bacteria to the harsh environment (Feiner et al., 2015; Argov et al., 2017; Keen and Dantas, 2018). For pathogens, these prophages are prominently related to the pathogenicity of its host (Feiner et al., 2015; Argov et al., 2017). Are prophages phiRv1 and phiRv2 and their phage proteins related to the pathogenicity of *M. tuberculosis*? Our previous study found that all sequenced strains of *M. tuberculosis* contained at least one prophage in the genome (Fan et al., 2014, 2016). And prophages do not exist in the *M. smegmatis* genome. Proteomics studies showed that some prophage genes in *M. tuberculosis* were translated and had function (Deng et al., 2013; Schubert et al., 2013). The transcriptome or expression profile data of *M. tuberculosis* under various stresses were explored (Fan et al., 2016), and it was found that some prophage genes were up-regulated under different environmental stresses. They may act as virulence genes or as regulatory genes to help *M. tuberculosis* resist the harsh environment in macrophages and to improve the pathogenicity of *M. tuberculosis.*

This study focuses on *Rv2650c*, a prophage phiRv2 gene. There is not homolog of *Rv2650c* in the chromosome of *M. smegmatis* based on blastx. Transcriptome analysis revealed that it was up-regulated in both the *M. tuberculosis*-infected macrophage model and persister model (Fan et al., 2016). Ka/Ks value (1.33) of *Rv2650c* gene indicating that the *Rv2650c* gene was strongly positively selected in *M. tuberculosis*. It may play an important role in the evolution of *M. tuberculosis* from environmental saprophytic bacterium to intracellular pathogen, possibly encoding virulence proteins involved in intracellular survival of *M. tuberculosis.*

Western blot result showed that protein Rv2650c was localized on the cell wall of *M. smegmatis*. Further microbiological experiments found that its expression enhanced the ability of *M. smegmatis* to survive in macrophages and the resistance to acidity, oxidative stress, and surfactants. The result of the growth curve showed that the growth kinetics of MS_Rv2650c and MS_Vec were similar, which excluded the possibility that more bacterial cells help *M. smegmatis* increase resistance to adverse environments. Prophage is a temperate phage genome or its fragment that is inserted into the bacterial chromosome. During the evolution process, bacteria will discard some inactive prophage genes obtained and choose to store prophage genes that can help the host grow or survive in the harsh environment (Obeng et al., 2016). As a prophage gene, *Rv2650c* encoded a functional protein localized on the cell wall that helped the host bacteria to resist harsh environments. Further, ELISA experiments also confirmed that some pro-inflammatory cytokines such as TNF, IL-10, IL-1β, and IL-6 were inhibited by prophage protein Rv2650c. The expression of inflammatory cytokines in macrophages play an important role in killing and eliminating *M. tuberculosis* (Lee et al., 2003; Krishnan et al., 2013; Li et al., 2016). We speculated that the *M. tuberculosis* prophage gene *Rv2650c* can inhibit the expression of inflammatory factors TNF, IL-1β, and IL-6 in macrophages to reduce the killing of *M. smegmatis* and promote intracellular survival of bacteria in macrophages.

In short, we have initially discovered that the prophage gene *Rv2650c* is of great significance for the survival of *Mycobacterium*. In the future, we will continue to study the function of the prophage gene and explore its mechanism in order to better clarify the mechanism of prophage in *M. tuberculosis*. This study provides novel insights into the discovery of virulence factors, as well as important new information for the role of prophage in the infection mechanism of *M. tuberculosis* and also builds the foundation for developing new drugs to control *M. tuberculosis.*

## Data Availability Statement

The original contributions presented in the study are included in the article/Supplementary Material, further inquiries can be directed to the corresponding author/s.

## Author Contributions

XF and WL participated in the design of the study. XF and ZL analyzed the data and wrote the manuscript. ZL, ZW, HZ, MJ, and ZL helped to modify the manuscript. All authors read and approved the final manuscript.

## Conflict of Interest

The authors declare that the research was conducted in the absence of any commercial or financial relationships that could be construed as a potential conflict of interest.

## Publisher’s Note

All claims expressed in this article are solely those of the authors and do not necessarily represent those of their affiliated organizations, or those of the publisher, the editors and the reviewers. Any product that may be evaluated in this article, or claim that may be made by its manufacturer, is not guaranteed or endorsed by the publisher.

## Supplementary Material

Supplementary Figure 1 Map of plasmid pNIT-Myc-Rv2650c.

Supplementary Figure 2 Comparison of the morphology of the recombinant *M. smegmatis* MS_Rv2650c and MS_Vec. **(A)** MS_Rv2650c and MS_Vec strains were cultured in 7H9 medium with 0.05% (v/v) Tween 80 and 28 mM ε-caprolactam at 37°C until an OD_600_ of 0.6–0.8 were reached. The cultures were kept at room temperature for 30 min. **(B)** The cultures were harvested for scanning electron microscopy (SEM) analysis.
