# Supplementary figures and images for: Prophage Gene Rv2650c Enhances Intracellular Survival of Mycobacterium smegmatis
Source: Front Microbiol. 2022 Jan 17;12:819837. doi: 10.3389/fmicb.2021.819837 (PMC8801708; doi:10.3389/fmicb.2021.819837)

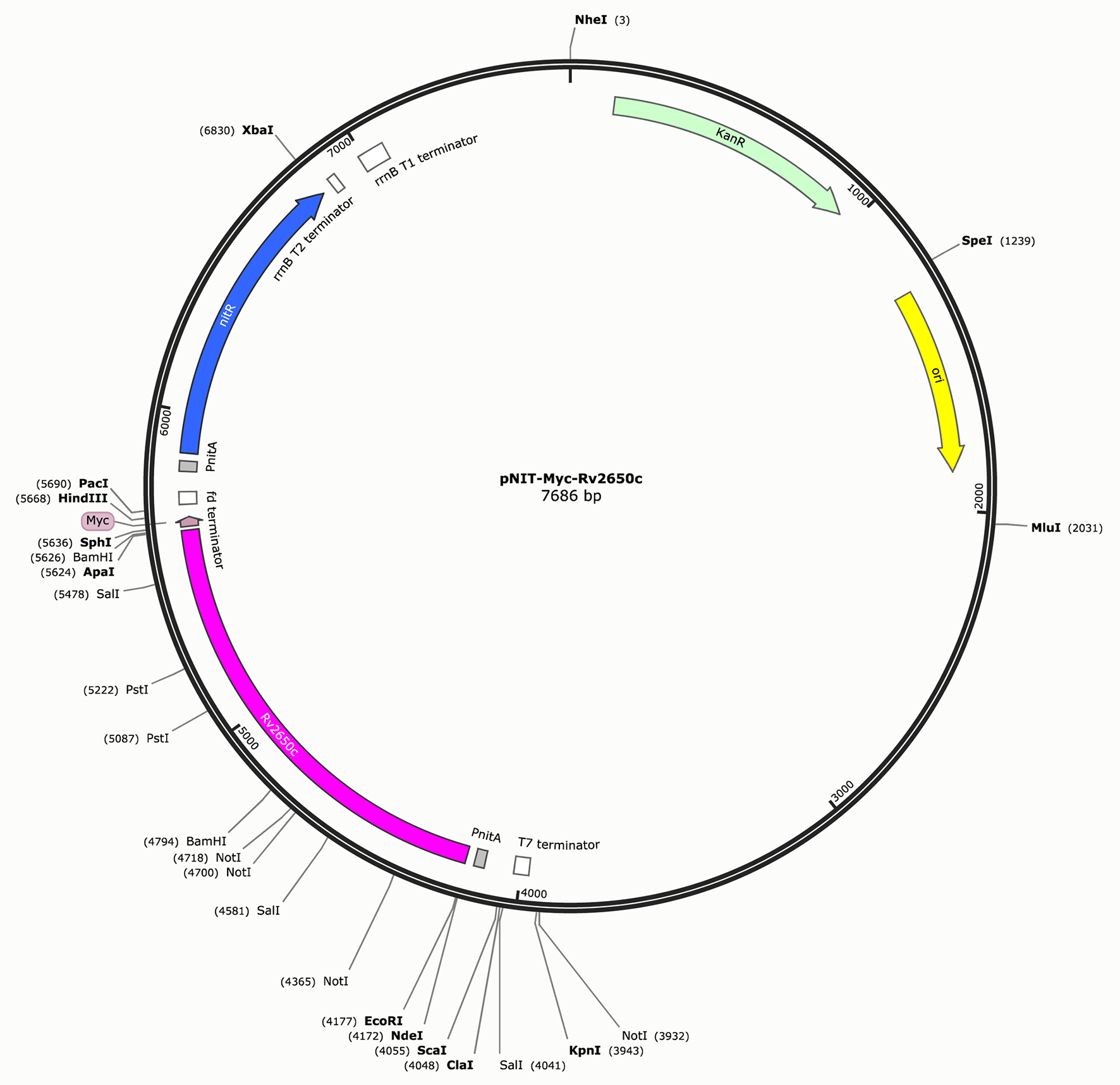

Supplement: Supplementary Figure 1 — Map of plasmid pNIT-Myc-Rv2650c. [file Image_1.TIF]

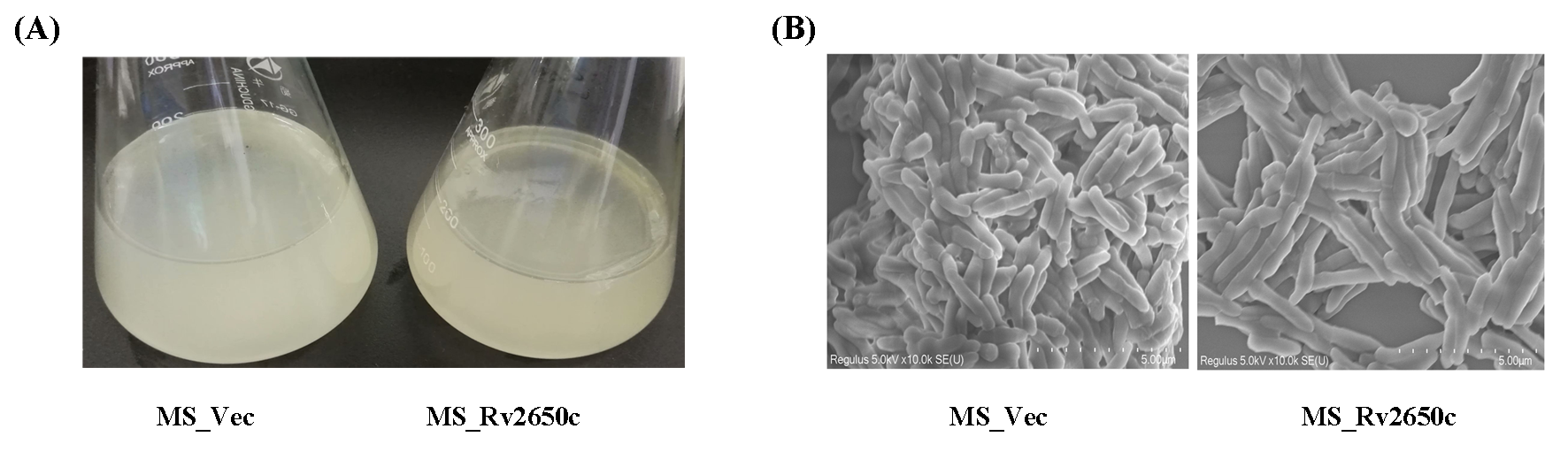

Supplement: Supplementary Figure 2 — Comparison of the morphology of the recombinant M. smegmatis MS_Rv2650c and MS_Vec. (A) MS_Rv2650c and MS_Vec strains were cultured in 7H9 medium with 0.05% (v/v) Tween 80 and 28 mM ε-caprolactam at 37°C until an OD600 of 0.6–0.8 were reached. The cultures were kept at room temperature for 30 min. (B) The cultures were harvested for scanning electron microscopy (SEM) analysis. [file Image_2.TIF]
